# Cell-Cell Adhesions and Cell Contractility Are Upregulated upon Desmosome Disruption

**DOI:** 10.1371/journal.pone.0101824

**Published:** 2014-07-09

**Authors:** Kaelyn Sumigray, Kang Zhou, Terry Lechler

**Affiliations:** Depts. of Dermatology and Cell Biology, Duke University Medical Center, Durham, North Carolina, United States of America; University of North Carolina at Chapel Hill, United States of America

## Abstract

Desmosomes are perturbed in a number of disease states – including genetic disorders, autoimmune and bacterial diseases. Here, we report unexpected changes in other cell-cell adhesion structures upon loss of desmosome function. We found that perturbation of desmosomes by either loss of the core desmosomal protein desmoplakin or treatment with pathogenic anti-desmoglein 3 (Dsg3) antibodies resulted in changes in adherens junctions consistent with increased tension. The total amount of myosin IIA was increased in desmoplakin-null epidermis, and myosin IIA became highly localized to cell contacts in both desmoplakin-null and anti-Dsg3-treated mouse keratinocytes. Inhibition of myosin II activity reversed the changes to adherens junctions seen upon desmosome disruption. The increased cortical myosin IIA promoted epithelial sheet fragility, as myosin IIA-null cells were less susceptible to disruption by anti-Dsg3 antibodies. In addition to the changes in adherens junctions, we found a significant increase in the expression of a number of claudin genes, which encode for transmembrane components of the tight junction that provide barrier function. These data demonstrate that desmosome disruption results in extensive transcriptional and posttranslational changes that alter the activity of other cell adhesion structures.

## Introduction

Desmosomes are robust cell-cell adhesion structures that provide mechanical integrity to tissues. They are particularly abundant in the epidermis and heart due to the significant mechanical stresses these tissues experience. The importance of desmosomes is highlighted by the many diseases associated with mutations in desmosomal genes. Depending on the gene affected and the severity of the mutation, symptoms can range from focal skin thickening and curly hair to lethal blistering disorders [1]. In addition, the autoimmune diseases pemphigus vulgaris (PV) and pemphigus foliaceus result from pathogenic antibodies against desmosomal cadherins [2], [3]. Finally, bacterial toxins target desmosomes in Staphylococcal scalded skin syndrome [4].

The response of cells and tissues to desmosome disruption is complex. In pemphigus, a number of studies have identified signaling pathways that become activated by pathogenic antibodies [5]–[9]. In addition, microarray analyses have revealed significant changes in transcript profiles upon treatment of human keratinocytes with PV sera [10]. However, previous studies have not examined in detail whether desmosome disruption affects adherens junction or tight junction activity, the other two prominent cell-cell adhesion structures in the epidermis. Furthermore, we do not know whether the responses to genetic disruption of desmosomes are similar to those elicited by autoimmue disruption.

In addition to desmosomes, both cultured keratinocytes and the epidermis have robust adherens junctions and tight junctions. Adherens junctions are structurally similar to desmosomes, and some studies have demonstrated a requirement for adherens junctions in desmosome and tight junction formation [11]–[15]. In addition, loss of desmoplakin resulted in changes in the morphology of both adherens junctions and the actin cytoskeleton in cultured keratinocytes [16]. However, the functional status of adherens junctions was not further investigated. In contrast, overexpression of the head domain of desmoplakin in A431 cell resulted in desmosome defects, but no overt adherens junction defects were reported [17]. While desmosomes and adherens junctions provide mechanical integrity and adhesion strength to the epidermis, tight junctions are essential for the barrier function of the skin [18]. Tight junction function has not been examined upon disruption of desmosomes.

In addition to their role in binding keratins, we previously reported that desmosomes are required for microtubule reorganization during epidermal differentiation. The reorganization of microtubules to the cell cortex has two important effects: 1) it strengthens adherens junctions through forces generated by myosin II, 2) it increases tight junction barrier activity [19]. Because of these previous findings, we wanted to determine whether loss of desmosomes resulted in changes in the composition, expression or function of these other important cell adhesion structures. Alterations in these could either exacerbate or ameliorate the effects of loss of desmosomes, and thus, may be important diagnostically and/or therapeutically.

## Results

### Myosin II-dependent changes in adherens junctions in desmoplakin-null cells

**Figure 1:**
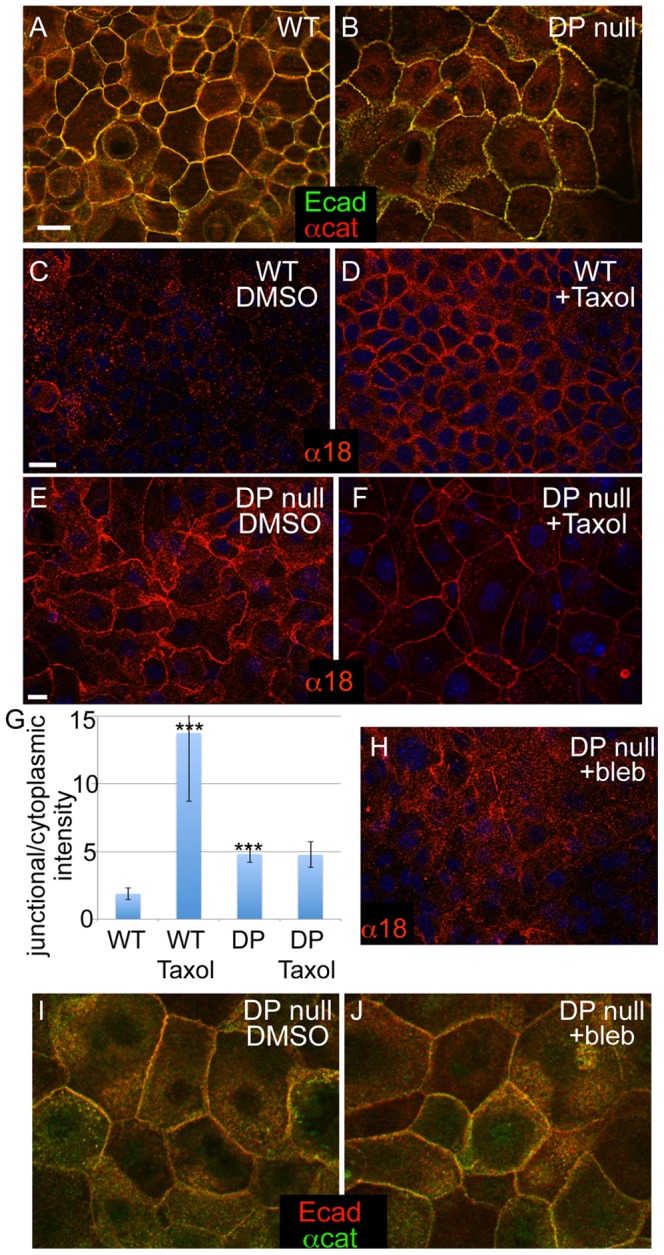
Changes in adherens junctions in DP-null keratinocytes. (A-B) WT and DP-null keratinocytes stained for E-cadherin (green) and α-catenin (red) after 24 h in Ca^2+^. (C-F) WT (C,D) and DP-null (E,F) keratinocytes stained with the tension-sensitive anti-α-catenin antibody, α18 (red). The α18 epitope is exposed in WT keratinocytes after microtubules are reorganized to the cell cortex with taxol treatment, 1 hour at 10 µM. (D). DP-null keratinocytes have the α18 epitope exposed at steady state (E). The junctional intensity is not significantly changed upon treatment with taxol (F). (G) Quantitation of junctional/cytoplasmic intensity of α18 in indicated samples. n>100 cells from at least two independent experiments. p values as compared to WT DMSO are <0.0001 for both WT with taxol and DP KO DMSO. There was no significant difference between DP KO DMSO verses taxol. (H) α18 (red) staining of DP null keratinocytes treated with the myosin II inhibitor blebbistatin (25 µM for 1 hour). (I,J) E-cadherin (red) and α-catenin (green) localization in DP cells treated either with DMSO (I) or 25 µM blebbistatin (J) for 1 hour. Scale bar, 10 µm.

To better understand the cellular and tissue responses to loss of desmosomes, we began by examining the status of adherens junctions in desmoplakin-null mouse keratinocytes. We found that both the transmembrane protein E-cadherin and the peripheral membrane protein α-catenin were localized to cell-cell junctions in both wild type and desmoplakin-null keratinocytes grown in the presence of 1.2 mM calcium for 24 hours (Fig 1A,B). Although adherens junction components were present at cell-cell contacts, the organization of those contacts was distinct in the desmoplakin-null cells. Rather than having junctions that formed relatively straight lines, the localization of adherens junction proteins in desmoplakin-null cells was more punctate, reminiscent of their localization during adherens junction assembly. This phenotype was seen in three independently derived DP null keratinocyte lines. The adherens junction phenotype in these cell lines was similar to, but somewhat less dramatic, than that previously reported in primary mouse keratinocytes [16]. In addition, localization of E-cadherin and other adherens junction proteins was normal in intact desmoplakin null epidermis [16]. Thus, loss of desmoplakin does not result in a significant impairment of the localization of adherens junction components to cell-cell contacts, although it may affect either their maturation and/or their morphology.

Our previous data demonstrated that desmosomes are essential for microtubule reorganization in keratinocytes [19], [20]. The reorganization of microtubules to the cell cortex resulted in changes in adherens junctions that were consistent with them being under tension. One marker that we and others have used to examine the tension status of the adherens junction is the α18 antibody [19], [21]. This antibody recognizes an epitope of α-catenin that is not accessible under low-tension conditions, but becomes exposed when the adherens junction is under tension [21]. Additionally, exposure of this epitope is lost upon myosin II inhibition, further suggesting that this is due to tension [19], [21]. That said, it is important to note that this antibody recognizes a conformation of α-catenin that could alternatively be induced independent of tension. As one marker of adherens junction status, we therefore examined the exposure of the α18 epitope in desmoplakin null cells. To our surprise, we found that this epitope was always exposed in desmoplakin-null cells, in contrast to its induced exposure by increasing tension (through taxol treatment) in wild type cells (Fig 1 C-E). Quantitation of the relative cortical/cytoplasmic intensity demonstrated that the enrichment of α18 at the desmoplakin-null cell cortex was significantly higher than the wild type control, but lower than the wild type cells treated with taxol. As expected, taxol treatment did not cause a further increase in cortical α18 levels in desmoplakin null cells. This is consistent with our previously published work demonstrating that desmoplakin null cells cannot reorganize their microtubule to the cell cortex [19], [20]. To determine whether the exposure of the α18 epitope was indeed myosin II-dependent in this context, we treated DP-null cells with the myosin II inhibitor blebbistatin. Inhibition of myosin II resulted in a decrease in cortical staining with the α18 antibody, indicating that myosin II-dependent forces act on adherens junctions in the absence of functional desmosomes (Fig 1E, F). Blebbistatin treatment did not demonstrably alter the localization of E-cadherin or α-catenin, so the effects are unlikely to be secondary to junctional reorganization (Fig 1I, J). These data suggest that DP-null cells have a higher degree of junctional contractility at steady state compared to WT cells.

### Myosin II is upregulated in desmoplakin-null epidermis

**Figure 2:**
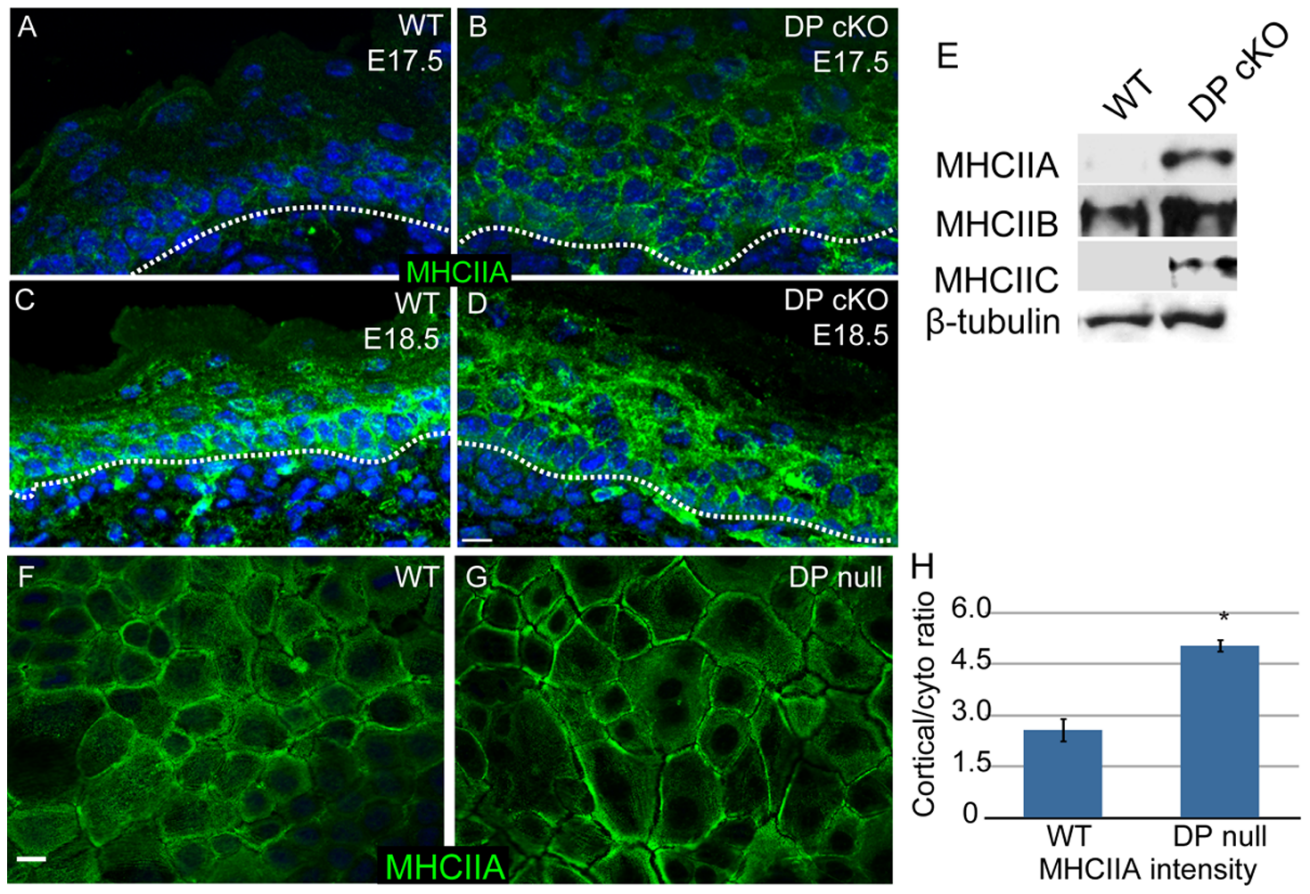
Myosin II levels and localization are upregulated in DP-null cells and epidermis. (A-D) WT (A, C) and DP cKO (B, D) epidermis at E17.5 (A, B) and E18.5 (C, D) were stained for myosin IIA (green). Basement membrane is marked with a dashed line. Scale bar, 10 µm. (E) Myosin II levels in E18.5 WT and DP cKO epidermis were evaluated by Western blot. β-tubulin is the loading control. (F, G) WT and DP-null keratinocytes were stained for myosin IIA (green). (H) Quantitation of cortical/cytoplasmic ratios of myosin IIA in WT and DP null cells. n>60 cells for two independent experiments, p = 0.027.

To understand this change in adherens junction status, we began by examining the levels and localization of type II myosins in intact epidermis and in cultured cells. In the WT epidermis, myosin IIA levels were low at embryonic day (e) 17.5 and increased by e18.5 (Fig 2A, C). In contrast, myosin IIA was easily detectable at e17.5 and was present at elevated levels at e18.5 in DP cKO epidermis (Fig 2B, D). This was especially evident in the suprabasal epidermis where desmosomes are most abundant. Western blot analysis of epidermal lysates confirmed an increase in the total amount of myosin IIA in the mutant epidermis at e18.5 (Fig 2E). This change was not specific to myosin IIA, as both myosins IIB and C were present at higher levels in the DP cKO epidermis (Fig 2E). We next examined myosin IIA localization in cultured WT and DP-null keratinocytes to get a higher resolution view of its subcellular organization. We found an approximately 2-fold increase in the levels of cortical myosin IIA in DP-null cells compared to WT cells (Fig 2F-H), which is consistent with increased cortical contractility and increased tension on adherens junctions.

### Treatment with pathogenic pemphigus antibodies results in increased recruitment of myosin II to the cortex

**Figure 3:**
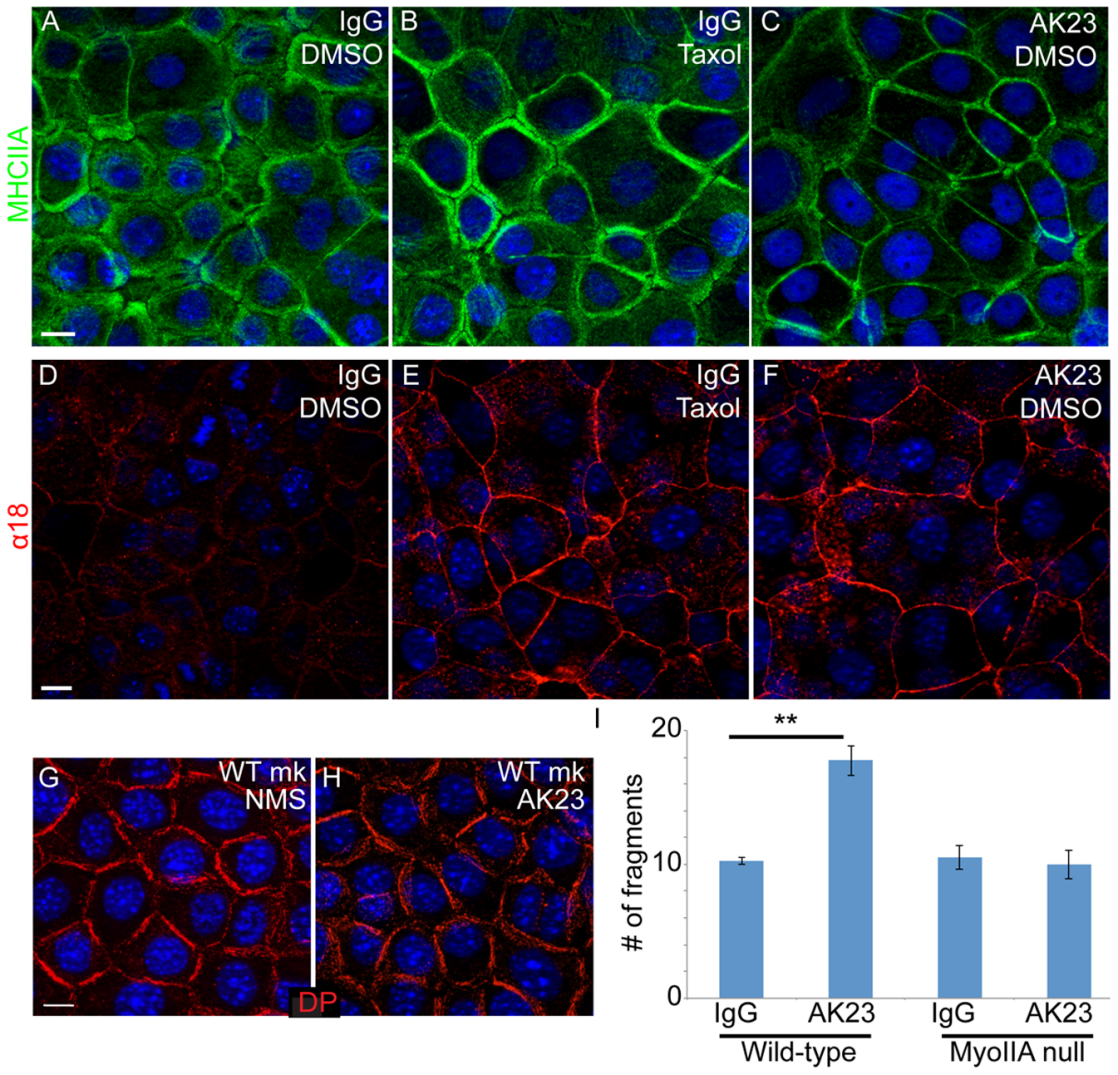
Pathogenic pemphigus antibodies induce increased contractility in WT keratinocytes. (A-C) WT keratinocytes were stained for myosin IIA (green). Cortical myosin II staining is observed when cells are treated with taxol to increase tension (B), or when treated with pathogenic pemphigus antibodies (AK23, in C). (D-F) WT keratinocytes were stained for α18 (red). The tension-sensitive epitope of α-catenin is exposed after taxol treatment (E) or after pathogenic pemphigus antibody treatment (F). (G,H) Desmoplakin (red) localization in WT cells treated with normal mouse sera (G) or with AK23 antibodies (H). Scale bars, 10 µm. (I) WT and myosin IIA-null keratinocytes were subjected to cell sheet disruption after treatment with control IgG or pathogenic pemphigus antibody. **, p<.005, n = 4.

The changes in myosin IIA levels and localization could either be due to specific loss of desmoplakin or a general response to desmosome perturbation. To test this, we examined the effects of desmosome perturbation by treatment with the monoclonal pathogenic anti-Dsg3 antibody, AK23 [22]. Because treatment times were relatively short, we examined the localization but not the total levels of myosin IIA. Similar to the loss of desmoplakin, AK23 treatment resulted in increased association of myosin IIA with cell junctions (Fig 3A-C). This closely resembled the myosin IIA localization in DP-null cells or in WT cells with cortical microtubules. However, it was not due to loss of cortical desmoplakin, as, consistent with a previous report [23], we found no significant effect of AK23 treatment on desmoplakin localization (Fig 3G-H). Therefore, perturbation of desmosomes by two distinct methods resulted in changes in myosin II localization.

The increased association of myosin II with cell-cell junctions upon AK23 treatment was particularly interesting, as it suggested that contractility may play a role in the disease pathology of pemphigus. However, the presence of myosin II at the cortex of AK23-treated cells does not necessarily mean that adherens junctions were altered. We therefore examined whether the α18 epitope of α-catenin was exposed upon AK23 treatment. Staining with the α18 antibody revealed robust cortical exposure of the epitope after AK23 treatment (Fig 3D-F), demonstrating a change in adherens junctions' status upon pathogenic antibody treatment, similar to the effects of genetic loss of desmoplakin.

### Myosin IIA exacerbates pemphigus-induced tissue fragility

Increased myosin II contractility and engagement of adherens junctions could result in one of two disparate outcomes. First, this could be a protective effect – by upregulating adherens junction engagement and adhesion, cells could compensate for the loss of desmosome-mediated adhesion. This would suggest that loss of myosin contractility would exacerbate the effect of pemphigus antibodies, resulting in increased tissue fragility. Alternatively, in the absence of robust desmosomes, the increase in tension provided by myosin II contractility could promote cell separations by physically pulling cells apart from one another. Thus, the loss of myosin II would prevent or decrease fragility induced by pemphigus antibodies. To distinguish between these possibilities, we treated WT and myosin IIA-null keratinocytes with control or pathogenic pemphigus antibodies. Mutant cells showed no defect in cell sheet strength under control conditions, as assayed by dispase-based cell sheet disruption assays. However, while AK23 antibodies caused an increase in the fragility of WT keratinocytes, myosin IIA-null keratinocytes were protected from AK23-induced fragility (Fig 3I). These data suggest that myosin IIA activity promotes tissue fragility in response to desmosome disruption. However, it is also possible that myosin II works in a non-canonical fashion to promote cell separations.

### Tight junction changes upon desmosome disruption

**Figure 4:**
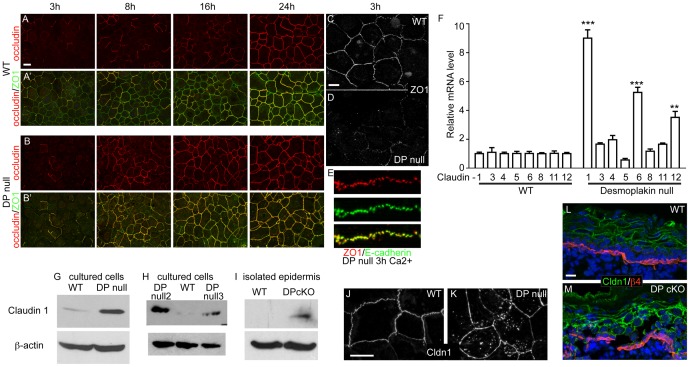
Tight junctions are altered upon loss of desmoplakin. (A-B′) Calcium was added to WT and DP-null keratinocytes, and cells were fixed at various time points and stained for tight junction proteins occludin (red) and ZO-1 (green). Scale bar, 10 µm. (C,D) ZO-1 staining of WT and DP-null keratinocytes at 3 hour after calcium switch. (E) Co-stain for ZO-1 (red) and E-cadherin (green) in DP null keratinocytes 3 hours after calcium switch. (F) RNA was isolated from WT and DP-null keratinocytes, and RT-PCR for several claudins was performed. ***, p<.0005, **, p<.005. n = 3. (G-I) Western blot analysis of total levels of claudin-1 and β-actin in lysates from cultured keratinocytes (G,H) and from isolated epidermis (I). (J,K) Claudin 1 staining of WT and DP-null keratinocytes 24 hours after calcium shift. (L,M) Immunofluorescence analysis of claudin 1 (green) and β4-integrin (red) in wild type (L) and desmoplakin conditional null epidermis (M). Scale bars are 10 µm.

In cultured keratinocytes, the tension-induced engagement of adherens junctions leads to an increase in tight junction-dependent barrier activity [19]. To determine whether this pathway was functional in DP-null cells, we began by examining the localization of two tight junction proteins in a calcium shift assay in which differentiation and cell adhesion are initiated by the addition of 1.2 mM calcium to the medium. Two interesting differences between the WT and DP-null keratinocytes were noted. First, there was a delay in ZO-1 localization to cell-cell contacts in the DP-null cells (Fig 4 A-D). This was noted in all three cell lines generated. However, by 8 hours after calcium addition, the localization of ZO-1 was similar to the WT controls (Fig 4A′-B′). Early ZO-1 localization to the cell cortex is likely due to its association with adherens junctions [24], [25], and we noted co-localization between E-cadherin and ZO-1 at these early time points (Fig 4E). While ZO-1 localized to cell-cell adhesions early, the transmembrane protein occludin was only sparsely found at cell-cell adhesions at early time points (Fig 4A). In WT cells, it became more broadly localized to cell junctions by 24 hours after calcium addition. In contrast, there was a clear increase in the kinetics of the association of occludin with the membrane in DP-null cells, and junctions were uniformly labeled by 16 hours after calcium addition compared to 24 hours in WT cells (Fig 4B-B′). Quantitation at 16 hours revealed that 94+/−4% of WT ZO1 junctions were occludin positive, while only 44+/−9% of KO (3 independent experiments, n>100 for each).

### Loss of desmoplakin results in transcriptional changes of claudins

Tight junction are regulated in various ways, including the expression levels of distinct claudins, transmembrane components of the tight junction that are necessary for tight junction strand formation [26]. We began by examining the mRNA levels of various claudins that have previously been reported to be expressed in the epidermis under normal or pathogenic conditions. We found significant increases in three of the eight claudin genes we examined – Claudins 1 (9X), 6 (6X) and 12 (4X) (Fig 4F). Not only was claudin-1 mRNA increased in DP-null cells, but the level of the protein was also significantly higher in mutant versus wild type control lysates (Fig 4G). These changes in claudin-1 levels were found, to varying degrees, in the two additional independently generated desmoplakin null cell lines (Fig 4H). Therefore, an increase in claudin-1 levels was a reproducible effect of loss of desmoplakin. Immunofluorescence analysis demonstrated that desmoplakin null keratinocytes had increased intracellular vesicular pools of claudin-1 as compared to the wild type controls (Fig 4J,K).

When we tested tight junction function by measuring transepithelial resistance, we found that different desmoplakin null keratinocyte lines had significantly different TER levels. While two cell lines had TER values near wild-type levels (250 Ωcm^2^) one desmoplakin null line had TER approximately 10X wild-type levels (2500 Ωcm^2^). Therefore, loss of desmoplakin is not inconsistent with functional TER, however, this occurs in the absence of mechanical force. It is important to note that desmoplakin null epidermis is severely disrupted and therefore clearly does not maintain barrier function with intact tight junctions. This is likely due to mechanical stresses in intact epidermis that are absent in cultured cells.

We next wanted to determine whether the changes in levels of claudin-1 also occurred *in vivo*, to determine whether this might be a marker for desmosome dysfunction in intact tissue. By immunofluorescence analysis, we noted an increase in intensity of claudin-1 staining (Fig 4L,M), which we confirmed by western blot analysis of lysates prepared from WT and DP-null suprabasal epidermis (Fig 4I). Therefore, both *in vitro* and *in vivo*, desmoplakin loss resulted in increased levels of claudin-1.

## Discussion

Desmosomes are the principle structure in the epidermis that protects the tissue from mechanical stresses. Our data have shown that perturbation of desmosomes results in robust changes in other cell-cell junctions in the epidermis. In addition, desmosome disruption results in increased myosin II levels/cortical localization that may increase junctional contractility and exacerbate blistering disease phenotypes. As all these experiments were conducted with mouse tissues and cells, it will be important to determine whether similar changes occur upon disruption of desmosomes in humans.

These studies were initiated because of our earlier findings on the role of desmosomes in promoting microtubule reorganization, and the effect that microtubules have on adherens and tight junctions [19], [20]. Contrary to our expectations, we did not find loss of myosin II localization and/or adherens and tight junction function in desmoplakin null cells. Rather, both the establishment of cortical microtubules (which is desmosome-dependent) and the loss of desmosomes resulted in some similar effects, though the underlying mechanisms are clearly distinct. This suggests that robust pathways control cortical contractility and cell adhesion in both physiological and pathological situations.

Adherens junctions are known mechanosensors and mechanotransducers, able to change activity and composition under tension [21], [27]–[29]. These changes require myosin II-mediated contractility [21], [30], [31]. We expected that loss of desmosomal adhesion would result in loss of tension across adherens junctions in keratinocytes, as desmosomes provide the majority of the strength to resist mechanical stress. Surprisingly, we report evidence for an increase in contractility and adherens junction engagement upon desmosome disruption. This data was based, in part, upon the exposure of the α18 epitope of α-catenin. While this has been a useful surrogate for adherens junction tension, it may also reflect non-tension based changes in adherens junctions. Therefore, future biophysical approaches will be required to determine the functional status of these junctions. This is complicated by the concurrent changes in other adhesion structures. The changes in adherens junctions were associated with an increase in myosin II protein levels and increased cortical localization of myosin II, presumably due to signaling pathways activated in the absence of functional desmosomes. We propose that forces generated by the increased levels of cortical myosin II can promote cell-cell separations upon desmosome disruption. This suggests an active cellular involvement in cell separations as well as passive effects from external forces. Complete desmosome disruption by loss of desmoplakin is clearly sufficient to induce fragility. However, we propose that increased cell fragility under conditions where desmosomes are partially perturbed is promoted by myosin II function. At very low levels of perturbation it is possible that myosin II contractility actually aids cell adhesion, by promoting adherens junction engagement. However, when desmosomes are not strong enough to withstand the increased forces, this would result in overt cell separations. That said, it is important to note that the α18 antibody used in these studies reports on a conformational change of α-catenin. While this change is induced by tension, we cannot rule out that non-tension based pathways can result in similar conformational changes.

Our data also demonstrated an increase in the expression of a number of tight junction components in desmoplakin null keratinocytes, especially claudin-1. Desmoplakin null epidermis showed a similar increase in claudin-1 levels. While there have not been reports of tight junction activity in pemphigus patients, it is noteworthy that an upregulation of claudin 1 was found in Hailey-Hailey disease [32]. Hailey-Hailey disease results in blistering of the epidermis and is caused by a mutation in the ATP2C1 gene, resulting in desmosomal defects. This supports the idea that the upregulation of claudins may be a general response to loss of desmosome adhesion. Whether this is a response to increase myosin II and tension, or whether these changes are downstream of other signals is not yet known. In addition, the role these changes play in disease pathology is unclear. It is unlikely that claudin upregulation upon severe desmosome disruption is able to promote barrier formation, as the epidermis is so mechanically fragile. However, in less severe cases, this may increase barrier function to compensate for integrity defects. It is also of note that claudin-1 is present throughout the suprabasal epidermis, not just where functional tight junctions form. It is therefore possible that tight junction-independent functions for claudin-1, including promoting adhesion, also occur. However, at least in cultured DP null keratinocytes, not all of the claudin-1 makes it to cell junctions, and an increase in cytoplasmic/vesicular pools is observed. While genetic loss of function studies have demonstrated an essential role for claudin-1 in the epidermis [18], similar studies have not been performed for claudins 6 and 12. Surprisingly, overexpression of claudin 6 alone in differentiated epidermis resulted in differentiation and barrier defects, demonstrating that claudins can have unexpected roles on epidermal function [33]. Importantly, cultured desmoplakin knockout cells did not have defects in transepithelial resistance, suggesting that in the absence of mechanical forces, desmosomes are not necessary for this function of tight junctions.

One of the most interesting questions raised by this study is the nature of what cells and tissues are sensing when desmosomes are disrupted. Clearly, a number of signaling pathways are activated upon treatment with pemphigus sera, but it is not clear whether any of these also respond to genetic disruption of desmosomes. In theory, the cell could sense changes in signal transduction pathways controlled by desmosomes, the organization of the cytoskeleton that is altered upon desmosome disruption, or cell-cell adhesion through contractile mechanisms. Whether there is any mechanical sensing and signaling in response to pemphigus antibodies is an important question that will require further investigation. In this way, myosin II may be important for both sensing defects in adhesion as well as participating in the effects of desmosome disruption. Finally, whether these changes that we have identified or the signaling pathways that lead to them can be clinically targeted to ameliorate the effects of desmosome perturbation is an important outstanding question.

## Materials and Methods

### Cell culture and Mouse Studies

All studies were performed on mouse tissue and mouse keratinocytes. All animal studies were performed with approval from the Duke Animal Use and Care Program. Keratinocytes were isolated from the backskin of e18.5 embryos by dispase treatment and trypsinization. After several passages on fibroblast feeders, keratinocyte lines were grown in the absence of feeders in E low calcium media (3∶1 DMEM:F12 (Invitrogen) with insulin (Sigma, 0.5 µg/ml), cholera toxin (ICN Biomedicals, 0.1 nM), transferrin (Sigma, 0.5 µg/ml), hydrocortisone (Calbiochem, 0.4 µg/ml) and T3 (Sigma, 0.2 µM) with 15% FBS (Hyclone), which was chelated in Chelex (BioRad) to remove calcium. Calcium was supplemented to 0.05 mM with CaCl_2_. Three independent DP null keratinocyte lines were developed. At least two different lines were used for each experiment, and in some cases all three (as stated in text). For experiments, keratinocytes were grown to confluency and calcium was added to 1.2 mM to induce adhesion. For drug experiments, 24 hours after calcium switch, cells were treated with 10 µM blebbistatin (Sigma, an inhibitor of myosin II) or 10 µM taxol (Sigma, a microtubule stabilizer) for 1 hour. Cells were treated with 2 µg anti-Dsg3 antibodies (AK23, Medical and Biological Laboratories) for 4 hours before examination (added to cells with intact junctions). AK23 is a pathogenic mouse-anti-Dsg3 antibody [34], [35].

### Immunofluorescence

The following antibodies were used: rat anti-E-cadherin (1∶50; gift from Colin Jamora, Bangalore, India), rabbit anti- α-catenin (1∶100; Sigma), mouse anti-β-catenin (1∶100; Sigma), rat anti- α18 (1∶100; gift from Akira Nagafuchi, Kumamoto University, Japan), rabbit anti-myosin IIA, IIB and IIC (all 1∶100 for immunofluorescence, 1∶1000 for Western blot; all from Covance), rabbit anti-ZO1 (1∶100; Invitrogen), rabbit anti-occludin (1∶200; Abcam), and goat anti-claudin 1 (1∶200 for Western blot; Santa Cruz Biotech).

Keratinocytes were grown on glass coverslips in E low calcium media until confluent. Cells were induced to differentiate and form junctions by the addition of calcium to 1.2 mM. Twenty-four hours after addition of calcium, cells were fixed in either 4% paraformaldehyde in PBS +0.2% Triton X-100 for 8 minutes or in -20°C methanol for 2 minutes. They were blocked with 3% BSA, 5% NGS, and 5% NDS in PBS+0.2% Triton X-100 for 15 minutes. Primary antibody was incubated for 15 minutes, and secondary antibody was incubated for 10 minutes. Coverslips were mounted in a solution of 90% glycerol in PBS with 2.5 mg/ml *p-*Phenylenediamine (Sigma-Aldrich). Samples were imaged on a Zeiss AxioImager Z1 microscope with Apotome attachment and MRm camera. Junction/cytoplasm intensity was quantified in ImageJ. For each image two random regions of the cytoplasm were used to determine background fluorescence levels. Cortical fluorescence was determined by taking the mean of the fluorescence intensity over the cell junction and subtracting the background (cytoplasmic) levels.

### Western Blotting

The backskin of e18.5 mice was incubated with Dispase II (1.2 U/ml in PBS) overnight at 4°C to isolate the epidermis. The epidermis was minced and boiled in Laemmli sample buffer.

### TER

TER was measured as previously reported [19]. Briefly, cells were grown to confluence on Transwell inserts (Corning), calcium was added to 1.2 mM and transepithelial resistance was measured 48 hours after calcium addition with a MilliCell ERS-2 V-ohm-meter (EMD Millipore).

### Cell sheet integrity assays

Cell sheet integrity was measured as previously reported [19]. Briefly, mouse keratinocytes were grown in 6-well dishes to confluency, then calcium was added to 1.2 mM. 24 h after calcium addition, cells were treated with 4 µg antibody for 4 h, then dispase II (1.2 U/ml in PBS) was added to lift the cells off of the dish. The cell sheets were subjected to mechanical disruption by pipetting up and down 10 times. The number of sheet fragments was counted manually.

### Gene Expression Analysis

We extracted total RNA from WT and DP-null keratinocytes with the Qiagen RNeasy mini kit and used the SuperScript First-Strand Synthesis System (Invitrogen) to generate cDNA. Quantitative-comparative RT-PCR was performed on a StepOne Plus Real-Time PCR System (Life Technologies) using SYBR green reagent (SensiMix SYBR and Fluorescein Kit; Bioline). The average of the wild-type levels was set to 1 and levels of mRNA from the mutant were relative to this. All primer sequences for claudin genes assayed were previously validated (http://pga.mgh.harvard.edu/primerbank/). Cldn1 5′-ggggacaacatcgtgaccg and 5′-aggagtcgaagactttgcact, Cldn3 5′-accaactgcgtacaagacgag and 5′-cagagccgccaacaggaaa, Cldn4 5′-gtcctgggaatctccttggc and 5′-tctgtgccgtgatgttg, Cldn5 5′-gcaaggtgtatgaatctgtgct and 5′-gtcaaggtaacaaagagtgcca, Cldn6 5′-atggcctctactggtctgcaa and 5′-gccaacagtgagtcatacacctt, Cldn8 5′-gcaacctacgctcttcaaatgg and 5′-ttcccagcggttctcaaacac, Cldn10 5′-cgaatgagaaagtgaccaccc and 5′-attagtcctctacatgcctggat, Cldn11 5′-atggtagccacttgccttcag and 5′-agttcgtccatttttcggcag, Cldn12 5′-tgtccttcctgtgtggtattgc and 5′-aaatcgtcaggttcttctcgttt, Cldn18 5′-ccgccgtgttccagtatgaag and 5′-cgatcatcagggctcgtacag.

### Statistical analysis

Statistical analyses were performed in GraphPad Prism software. A student's t-test was used to determine statistical significance. P<0.05 was considered statistically significant.
